# Quantifying Habitual Physical Activity and Sedentariness in Older Adults—Different Outcomes of Two Simultaneously Body-Worn Motion Sensor Approaches and a Self-Estimation

**DOI:** 10.3390/s20071877

**Published:** 2020-03-28

**Authors:** Rieke Trumpf, Wiebren Zijlstra, Peter Haussermann, Tim Fleiner

**Affiliations:** 1 Institute of Movement and Sport Gerontology, German Sport University Cologne, 50933 Cologne, Germany; 2 Department of Geriatric Psychiatry & Psychotherapy, LVR-Hospital Cologne, 51109 Cologne, Germany

**Keywords:** actigraphy, hybrid motion sensors, physical activity, sedentariness

## Abstract

Applicable and accurate assessment methods are required for a clinically relevant quantification of habitual physical activity (PA) levels and sedentariness in older adults. The aim of this study is to compare habitual PA and sedentariness, as assessed with (1) a wrist-worn actigraph, (2) a hybrid motion sensor attached to the lower back, and (3) a self-estimation based on a questionnaire. Over the course of one week, PA of 58 community-dwelling subjectively healthy older adults was recorded. The results indicate that actigraphy overestimates the PA levels in older adults, whereas sedentariness is underestimated when compared to the hybrid motion sensor approach. Significantly longer durations (hh:mm/day) for all PA intensities were assessed with the actigraph (light: 04:19; moderate to vigorous: 05:08) when compared to the durations (hh:mm/day) that were assessed with the hybrid motion sensor (light: 01:24; moderate to vigorous: 02:21) and the self-estimated durations (hh:mm/day) (light: 02:33; moderate to vigorous: 03:04). Actigraphy-assessed durations of sedentariness (14:32 hh:mm/day) were significantly shorter when compared to the durations assessed with the hybrid motion sensor (20:15 hh:mm/day). Self-estimated duration of light intensity was significantly shorter when compared to the results of the hybrid motion sensor. The results of the present study highlight the importance of an accurate quantification of habitual PA levels and sedentariness in older adults. The use of hybrid motion sensors can offer important insights into the PA levels and PA types (e.g., sitting, lying) and it can increase the knowledge about mobility-related PA and patterns of sedentariness, while actigraphy appears to be not recommendable for this purpose.

## 1. Introduction

Sedentariness negatively impacts the health of older adults e.g., risk of chronic diseases, falls, and reduced quality of life [1,2,3,4,5,6]. Applicable and accurate assessment methods are required for a clinically relevant quantification of habitual physical activity (PA) levels and sedentariness in older adults.

Questionnaires, activity logs, or diaries are often used as self-estimations of PA levels. These are cheap instruments that allow for assessing a large number of participants [7], but they often fail to address sedentariness as well as activities with light intensity [8,9]. A recall-bias, especially regarding activities of daily living and over- or underestimation of PA, further limits the use of self-estimations in older adults [10,11].

Body-worn motion sensors allow for continuously monitoring and objectively quantifying PA [12] in terms of frequency, duration, and intensity, over long periods (e.g., days, weeks, month) [8]. A common sensor-based approach for assessing PA is actigraphy and the quantification of PA in counts per epoch [8,13]. Actigraphs are small, watch-like devices, which allow for an objective assessment of PA with minimal obtrusiveness [8]. Based on device- and population specific thresholds of the actigraphy data, the PA-levels are classified into sedentary, light, moderate, and vigorous intensities [8]. However, the detection of sedentariness based on an analysis of activity counts from wrist-worn actigraphy appears to be challenging. In a previous trial, a sensitivity of only 53% in the detection of sedentariness in a sample of healthy adults during different activities was obtained [14]. The authors concluded that the inter-person variability in wrist movements during sedentariness might result in misclassification [14]. Furthermore, actigraphy and analysis based on counts does not allow for quantifying the type of PA [15].

Recently used body-worn hybrid motion sensors, incorporating accelerometers, gyroscopes, and magnetometers allow for analyzing the type of PA (e.g., sitting, walking) as well as detecting different postures (e.g., seated, standing) and postural transitions [16].

Current methodological reviews discuss the (dis-)advantages of quantifying PA levels and sedentariness based on counts and the PA type [8,9,17]. However, there is no gold standard for the assessment of habitual PA yet, and the results from a comprehensive comparison of self-estimated PA levels and different sensor approaches to quantify differences in older adults are lacking. To be able to relate results on PA of different approaches, the aim of this study is to compare habitual PA levels and sedentariness assessed with two simultaneously body-worn motion sensors: (1) an actigraph, (2) a hybrid motion sensor approach and a PA questionnaire in healthy older adults.

## 2. Materials and Methods

This investigation was part of the ChronoSense project—a cross-sectional trial to investigate the use of body-worn motion sensors to quantify circadian chronotypes in older adults (DRKS00015069, German clinical trials register). The Ethics Committee of the German Sport University Cologne approved the study protocol (registration number 156/2017).

### 2.1. Participants

The participants were included to the project based on the following criteria: community-dwelling, age of 65 years or older, a score on the Mini-Mental Status Examination (MMSE) ≥ 24 [18,19], subjective health (self-reported), and written informed consent to the study procedures. Persons with any acute or severe mobility impairment, cardiovascular disorder, cognitive disorder, or neurological disease (assessed with the Functional Comorbidity Index (FCMI) [20]), which could interfere with functional mobility, were excluded.

The recruitment strategy included sending out emails with information brochures to local senior citizens’ networks and employees of a large municipal association in the Rhineland region in Germany, and word of mouth referrals. Furthermore, persons who expressed interest in participating in studies of the Institute of Movement and Sport Gerontology in the past were invited by email or telephone call. It was ensured that these test persons had not participated in any scientific experiment in the previous year.

### 2.2. Instruments

The self-estimation of PA levels was assessed while using the German Physical Activity Questionnaire 50+ (GPAQ 50+) [21], a self-administered questionnaire assessing older adults’ PA level per week. The participants were asked to estimate for how many hours they performed certain activities on average per week during the last four weeks. The questionnaire covers activities related to the categories household, gardening, leisure time, exercising, and voluntary work. Each of the activities is assigned to a metabolic equivalent of task (MET) [22]. The overall score of the GPAQ 50+ is based on the quantification of the activity level in MET hours: activity duration [h/week] × MET or the energy expenditure: activity duration [h/week] × MET × body weight [kg]. The GPAQ 50+ was administered prior to the sensor-measurements. Therefore, the self-estimated PA levels refer to an average week during the four weeks before and the sensor measurement period.

The wrist-worn MotionWatch 8 (Camntech, Cambridge, UK) was used for the actigraphy-based assessment of PA levels. The MotionWatch 8 (MW8) integrates a triaxial accelerometer (sample frequency up to 11 Hz), a light sensor, and an event marker button. The MW8 allows data collection for up to three months. The participants wore it on the wrist of their non-dominant hand. The data were collected in the triaxial mode with an epoch length of 60 s.

The Dynaport Move Monitor+ (McRoberts, Den Haag, NL) was used as hybrid motion sensor. The Dynaport Move Monitor+ (MM+) consists of a triaxial accelerometer, a triaxial gyroscope, a triaxial magnometer, a barometer, and a temperature sensor. Sample frequency of the accelerometer and gyroscope was 100 Hz. The MM+ allows for a collection of data for up to seven consecutive days. The MM+ was attached to the participants’ lower back, approximately 3 cm right to the fifth vertebra of the lumbar spine (L5) using waterproof self-adhesive fixing foil (Opsite Flexifix, Smith and Nephew, London, UK), enabling a consistent recording of PA. The participants were asked not to swim, have a sauna or take a bath during the measurement period. Furthermore, the participants were asked to wear both sensors continuously during the measurement period. Only sensor data of participants with six or more complete measurement days were included to ensure an assessment of habitual PA levels.

### 2.3. Data Collection

Sensor-data were collected over the period of one week, aiming to monitor the participants’ PA over 24 h without interruption on all seven days of the week. The measurement period started with an individual appointment in the laboratory in which the GPAQ 50+ was administered and the participants were equipped with the two sensors. Furthermore, a general questionnaire assessing the participants living situation (e.g., material status, income) as well as the health status (e.g., number and kind of chronic diseases) was administered. The sensors were removed from the participants’ bodies and special incidents during the measurement period, possibly interfering with the participants’ PA (e.g., acute illness) were noted, during a second appointment after the end of the measurement period. As the aim of the study was to compare habitual PA levels, the participants were asked to estimate whether the measurement period was usual in terms of their habitual PA. If they considered the measurement period as unusual, the participants were asked to specify whether their PA was higher or lower than usually. Finally, the participants indicated whether or not they had removed one or both the sensors during the measurement period and specified the period if this was the case.

### 2.4. Data Processing

The sensor data were processed while using the respective manufacturer’s own algorithms. The output of the MW8 (DayAnalysis, CamNtech, Fenstanton, UK) includes total counts per 60 s epochs. Landry and colleagues [23] used concurrent measurements of actigraphy and indirect calorimetry during activities of daily living (e.g., lying, sitting, standing, walking) to validate the use of MW8 activity counts for dissociating sedentary, light, and moderate to vigorous PA in healthy older adults. The optimal cut-points for sedentary (<1.5 METs), light (1.5–3.0 METs) and moderate to vigorous (>3 METs) intensity (as in the Compendium of Physical Activities [22]) were determined from Receiver Operating Characteristic (ROC). For a full description, see Landry and colleagues [23]. The derived cut-points were as follows: for sedentariness ≤178.5 counts per minute with a sensitivity of 78%, specificity of 70%, and an accuracy of 71%, and for moderate to vigorous intensity ≥562.5 counts per min. with a sensitivity of 40%, a specificity of 90%, and an accuracy of 69%. Light PA was determined as the activity level between the boundaries for sedentariness and moderate to vigorous PA (i.e., between 178.5–562.5 counts per min.). In the present study, the cut-points that were established by Landry and colleagues [23] were used to determine activity intensity. Average counts per minute were calculated for the description of overall PA level.

The output of the MM+ included PA type (walking, stair walking, cycling, shuffling, standing, sitting, and lying) plus an additional category not-worn, corresponding MET-values, activity duration, and number of steps per 60 s epoch. Van Hees and colleagues [24] developed a model for the analysis of MM+ (MoveMonitor, McRoberts, The Hague, NL, USA) data that combines the type of activity and its intensity for the prediction of energy expenditure. A standardized protocol comprising lying, sitting, standing, and walking was used to determine the best-fit linear equations between movement intensity (as assessed with an accelerometer) during each type of activity, and activity related energy expenditure (as assessed via indirect calorimetry). A next step then determined for each second the detected type of activity and the equation to be used for estimating energy expenditure. The resulting model for prediction of energy expenditure was validated in a respiration chamber. Within subjects, the variation in energy expenditure explained by the model was 81%. Between subjects, the prediction model explained 58% to 70% of the variation in energy expenditure. For the description of PA types, the total durations of PA types and total number of steps were calculated. To determine PA with light, moderate to vigorous intensity and sedentariness for the MM+ the same MET-based thresholds that Landry and colleagues [23] used to calibrate the cut-points for the MW8 were used: <1.5 METs for sedentary, 1.5–3.0 METs for light, and >3 METs for moderate to vigorous PA.

Self-estimated durations for light and moderate to vigorous activity were assessed by summing up the reported durations for each intensity. The durations of sedentariness cannot be derived from the GPAQ 50+. The same MET-based thresholds as the MM+ were applied to determine PA with light and moderate to vigorous intensity.

The average durations per day of light and moderate to vigorous PA intensities, as well as sedentariness were calculated for all three assessment methods.

### 2.5. Statistical Analysis

Statistical analysis was conducted with IBM SPSS Statistics 26.0 for Windows (International Business Machines, Armonk, NY, USA). Extreme values of more than three times the interquartile distance were identified using boxplots and excluded from further analysis. Normal distribution was examined with the Kolmogorov Smirnov test after the exclusion of extreme values. Analyses of variance with repeated measurements (ANOVAs) or Friedman-tests were performed to assess differences in PA with light and moderate to vigorous intensity between the three methods. If no sphericity was given, the Greenhouse–Geisser correction was used. Significant differences were examined with the Bonferroni post hoc test. As sedentariness was not assessed with the GPAQ 50+, differences in the assessment of sedentariness between the MW8 and the MM+ were examined while performing a *t*-test for paired samples or the Wilcoxon-Test. An alpha <0.05 was considered to be statistically significant.

## 3. Results

### 3.1. Participants

A total of 118 community-dwelling older adults were screened for eligibility. Twenty-three persons did not confirm to participation, 10 persons did not fit the inclusion criteria. Data collection was initiated with 85 persons. Two participants were excluded due to acute illness during the measurement period. The data of one participant who indicated that he was less active than usual during the measurement period were excluded from analysis. Nine participants had to be excluded due to missing data of the MW8 and six due to missing data of the MM+. Eight participants were excluded, because MM+ data of less than six complete measurement days were available (mostly due to battery issues). One participant did not wear the MM+ according to the instructions and was excluded. Finally, data of 58 participants were analyzed. Table 1 shows their characteristics.

### 3.2. Results on Physical Activity

Table 1 presents the results of the PA assessment. The average measurement duration of the MW8 was seven days. All of the participants reported to have worn the MW8 continuously over the measurement period. The average measurement duration of the MM+ was 6.9 days. Two participants (3.4%) reported to have reattached the MM+ once during the measurement period.

### 3.3. Physical Activity Intensities

**Figure 1:**
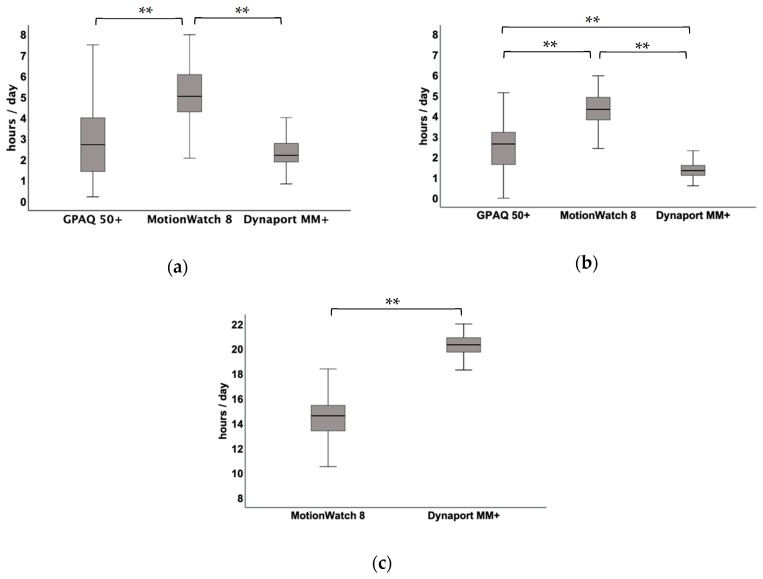
Box-plot illustration of German Physical Activity Questionnaire 50+, MotionWatch8 and MoveMonitor+ derived total duration [hour/day] of physical activity intensity and sedentariness: (**a**) moderate to vigorous intensity (Friedman-test with Bonferroni post-hoc test); (**b**) light intensity (repeated measurement analysis of variance with Bonferroni post-hoc test); and, (**c**) sedentariness (*t*-test for paired samples). ** *p* ≤ 0.01.

Figure 1a,b show the average durations of PA with light and moderate to vigorous intensities for the GPAQ 50+, MW8, and MM+ in hours per day. Figure 1c illustrates the duration of sedentariness that was assessed with the MW8 and the MM+. Two extreme values in the duration of PA with light activity assessed with GPAQ 50+ and the MM+ were identified and excluded from data analysis. Significant differences (p ≤ 0.01) in the durations of all PA intensities were found between all of the assessment methods, except for PA with moderate to vigorous intensity between the MM+ and the GPAQ 50+ (*p* = 0.412).

## 4. Discussion

The aim of this study was to compare habitual PA levels and sedentariness assessed with two simultaneously body-worn motion sensors, (1) an actigraph, (2) a hybrid motion sensor approach and a PA questionnaire in healthy older adults.

Longer durations for all PA intensities were assessed with the wrist-worn actigraph. Moreover, actigraphy-assessed durations of sedentariness were much shorter as compared to the durations that were assessed with the hybrid motion sensor. These results indicate that an actigraphy-based assessment of PA leads to an overestimation of older adults’ habitual PA levels when compared to a self-estimation and a body-worn hybrid motion sensor, as well as an underestimation of sedentariness when compared to a hybrid motion sensor.

The significantly longer durations of light (02:55 hh:mm/day), moderate to vigorous intensity (02:47 hh:mm/day) between the MW8 and the MM+, and the differences in durations of sedentariness (05:43 hh:mm/day) might be due to differences in sensor placement and data analyses. In case of the MW8, accelerometer raw data is analyzed to counts per epoch, which allows for assessing the presence or absence of activity and its intensity. Energy expenditure is predicted solely based on intensity, as determined from counts. Furthermore, the wrist-placement of the MW8 only allows for an assessment of hand and upper-limb activity. Information on body posture, acceleration, or movement direction and, thus, on mobility-related PA cannot be obtained with this method [25]. The analysis of the MM+ data and its lower-back placement provide, next to activity intensity and steps, information on the type of PA (e.g., shuffling, walking) and, thus, enables the assessment of mobility-related PA. The combination of information on the type of PA and its intensity is assumed to improve the prediction of energy expenditure, especially in sedentariness [24].

Previous findings also indicated that actigraphy-based assessment and an analysis based on activity counts lack sensitivity in the detection of sedentariness [8] and that the accurate assessment of sedentariness, that is based on wrist-worn accelerometer data, is challenging [14]. Nevertheless, the attachment of accelerometers to the hip/lower back has also been considered critical, due to problems with compliance and possible measurement error that is induced by changes in the sensor placement on the body [8,17]. In the trial of van Schooten and colleagues [15], the participants wore the MM+ in a belt around the hip, requiring them to remove it during (un-) dressing and activities, such as showering. In the present trial, we chose to attach the MM+ with waterproof, self-adhesive foil to the participants’ bodies to increase wearing times to a complete 24/7 schedule [25]. This attachment allows for also wearing the sensor during activities of daily living, like showering, makes it more difficult for the participants to take off the sensor themselves, and less likely to forget the reattachment of the sensor, as indicated by the low number of participants (n = 2) who reattached the sensor.

When comparing the MW8 and the MM+ regarding feasibility, the results indicate a somewhat better feasibility of the wrist-worn MW8 as compared to the lower back worn MM+ in the investigated sample of active healthy older adults. However, the results regarding the assessed durations of PA intensities and sedentariness and the detailed analysis options indicate the MM+ as the superior choice to the MW8.

The self-estimated durations for PA with light and moderate to vigorous intensity were significantly shorter when compared to MW8 and when compared to the MM+, the self-estimated durations of moderate to vigorous PA were significantly longer. Though these results seem to indicate that the GPAQ 50+ might be a useful tool for estimating durations of moderate to vigorous PA, the self-estimation of PA levels will always rely on the participant’s memory ability and it does not allow for a continuous quantification of habitual PA levels [8,9]. Therefore, its suitability appears to be limited, especially in the investigation of PA levels in patients with cognitive disabilities or the investigation of circadian aspects of PA.

This is the first study comparing the PA levels and sedentariness that were assessed with a simultaneously wrist-worn actigraph and a hybrid motion sensor-based approach. Large differences in the durations of PA intensities and sedentariness were observed between both sensor-based approaches. Actigraphy is based on the assessment of wrist movements and an analysis in counts per minute. Previous results indicate that an analysis based on wrist-worn actigraphy lacks sensitivity, especially in the detection of sedentariness [14]. Regarding the MM+, accuracies of 91–99% in the detection of sitting, and 97% in the detection of lying are reported [26]. Given these results, it can be assumed that wrist-worn actigraphy underestimates the durations of sedentariness in older adults. According to the Sedentary Behavior Research Network (SBRN), any waking behavior characterized by an energy expenditure ≤1.5 METs while in a sitting, reclining, or lying posture refers to sedentariness [27]. An analysis solely based on counts per minute does not allow for an evaluation of PA and sedentariness according to this definition [15]. Furthermore, more detailed information on sedentariness (activity type, frequency of postural shifts) is clinically relevant in understanding health outcomes [8,16]. Along with the growing evidence on sedentariness as an independent risk factor for diseases [8] and mobility-related PA as an important factor in the maintenance of health status and independent functioning [12,28], methods to quantify PA should be able to differentiate between sedentariness and mobility-related PA. Hybrid motion sensors, like the MM+, can provide this kind of information and should, therefore, be considered superior to actigraphy in the assessment of older adults’ habitual PA and especially sedentariness.

According to the guidelines of the American College of Sports Medicine (ACSM), older adults should achieve a PA level of 7.5 to 12.5 MET hours per week to maintain health status [29]. The investigated sample of older adults exceeds the ACSM recommendations, especially according to the self-estimation (GPAQ 50+ activity level of 145.2 MET hours). This difference might be due to the fact that the amount of PA that is recommended by the ACSM is in addition to routine activities of daily living (e.g., self-care, cooking, casual walking, shopping) [29]. The PA levels that were assessed with the GPAQ 50+, however, include these activities. The sensor-based assessments showed similar PA levels to those that were previously recorded in comparable samples. An average of 317.5 counts per minute assessed with the MW8 is in line with the results that were reported by Landry and colleagues (321.4 and 276.9 MW8 counts per minute; obtained from two simultaneously worn MW8s) [23]. Regarding the results of the MM+, an analysis of PA type showed a total duration of 09:57 hh:mm/day for lying, 08:47 hh:mm/day for sitting, 02:55 h for standing, 00:27 hh:mm/day for shuffling, and 01:50 hh:mm/day for walking per day. Van Schooten and colleagues [15] reported similar durations for the PA types. They observed a total duration of 09:48 hh:mm/day for lying, 09:46 hh:mm/day for sitting, 02:58 hh:mm/day for standing, 01:10 hh:mm/day for walking, and 00:12 hh:mm/day for cycling in a sample of healthy older adults aged 71 to 80 years.

There are methodological limitations regarding this study. First, of the initially 85 included persons, only the data of 58 participants were included in the analysis. We aimed to assess differences in the durations of PA with light, moderate to vigorous intensity, and sedentariness between the MW8 and the MM+. Therefore, only participants were included, for which data from both sensors were available. Second, we calculated the total durations of moderate to vigorous activity intensity for the three assessment methods, as the primary aim of this study was to compare the assessed PA levels and especially sedentariness. Traditionally, activities are considered to be moderate and vigorous activities when they were performed for a minimum of ten minutes without intermission. Thus, the present results regarding moderate to vigorous intensities are not comparable to previous results. Third, the self-estimated PA levels refer to the month prior to the sensor-measurements. We chose to use the GPAQ 50+ because its score refers to a usual week in terms of PA within the last four weeks, as the aim of this trial was to assess habitual PA levels in healthy older adults. Finally, the results regarding PA levels and sedentariness assessed with MW8 and the MM+ were based on proprietary algorithms, which may or may not fit well for all ages. Even though the activity classification for the MM+ was found to be valid when compared to observation in young and older adults [30], as well as patient populations [31], previous studies suggest that the differentiation between sitting and standing deserves improvement [32].

Taking into account that sedentariness is an independent risk factor for several health outcomes [1,2,3,4,5,6]; the results of this trial highlight the relevance of quantifying habitual PA levels in older adults. Sensor-based approaches can offer important insights into the PA levels and PA types (e.g., sitting, lying) of older adults and they can increase the knowledge about mobility-related PA and sedentariness patterns. Furthermore, information regarding mobility-related PA (e.g., number of steps, transitions) offers a broad basis of starting points for deriving personalized interventions to improve PA in older adults sustainably. The first findings indicate that PA monitor-based interventions are effective in increasing PA levels in older adults [33]. Future research is needed to evaluate how motion sensor data can be used as a basis for such interventions and as an individual feedback. Finally, the use of motion sensors to assess PA levels and develop and evaluate interventions aiming to increase PA and decrease sedentariness in older adults could be of great benefit for older adults and the health care system.

## Figures and Tables

**Table 1:** Sample characteristics and physical activity results of the German Physical Activity Questionnaire 50+, MotionWatch8, and the Move Monitor+.

			n (%)	Mean	SD	Min	Max
N			58				
Female			35 (60)				
Age				71.6	5.0	64	83
BMI				25.8	4.2	20	38
MMSE				28.8	1.3	25	30
Number of Diseases				2.1	1.4	0	7
FCMI				1.4	1.3	0	5
**Physical Activity**							
German Physical Activity Questionnaire 50+							
	activity level [MET hours/day]			145.2	88.9	22	423
	energy expenditure [kcal/week]			11193.6	7178.7	2903	38747
MotionWatch 8							
	counts / minute			317.5	82.0	135	563
Move Monitor +							
	activity duration [hh:mm/day]						
		lying		09:57	01:31	07:28	14:56
		sitting		08:47	01:47	04:52	12:59
		standing		02:55	00:45	00:57	04:35
		shuffling		00:27	00:07	00:10	00:47
		walking		01:50	00:34	00:41	03:02
		other activities*		00:04	00:09	00:00	00:59
	steps / day			9816.3	3539.6	3700	18321

BMI—Body Mass Index; FCMI—Functional Comorbidity Index (0–18 points; low scores indicate good functioning); h—hour; Kcal—kilocalories; m—minute; MET—Metabolic equivalent of task; MMSE—Mini Mental State Examination; SD—standard deviation; *—summation of total activity durations for cycling and stair walking.
